# Recurrent and concurrent patterns of regional BOLD dynamics and functional connectivity dynamics in cognitive decline

**DOI:** 10.1186/s13195-020-00764-6

**Published:** 2021-01-16

**Authors:** Lingyan Liang, Yueming Yuan, Yichen Wei, Bihan Yu, Wei Mai, Gaoxiong Duan, Xiucheng Nong, Chong Li, Jiahui Su, Lihua Zhao, Zhiguo Zhang, Demao Deng

**Affiliations:** 1 Department of Radiology, First Affiliated Hospital, Guangxi University of Chinese Medicine, Nanning, 530023 Guangxi China; 2 School of Biomedical Engineering, Health Science Center, Shenzhen University, Shenzhen, 518060 China; 3 Guangdong Provincial Key Laboratory of Biomedical Measurements and Ultrasound Imaging, Shenzhen, 518060 China; 4 Department of Acupuncture, First Affiliated Hospital, Guangxi University of Chinese Medicine, Nanning, 530023 Guangxi China; 5 Peng Cheng Laboratory, Shenzhen, 518055 China; 6 The People’s Hospital of Guangxi Zhuang Autonomous Region, Nanning, 530021 Guangxi China

**Keywords:** Mild cognitive impairment, Subjective cognitive decline, Dynamic functional connectivity, Default mode network, Fractional amplitude of low-frequency fluctuations

## Abstract

**Background:**

The brain’s dynamic spontaneous neural activity and dynamic functional connectivity (dFC) are both important in supporting cognition, but how these two types of brain dynamics evolve and co-evolve in subjective cognitive decline (SCD) and mild cognitive impairment (MCI) remain unclear. The aim of the present study was to investigate recurrent and concurrent patterns of two types of dynamic brain states correlated with cognitive decline.

**Methods:**

The present study analyzed resting-state functional magnetic resonance imaging data from 62 SCD patients, 75 MCI patients, and 70 healthy controls (HCs). We used the sliding-window and clustering method to identify two types of recurrent brain states from both dFC and dynamic regional spontaneous activity, as measured by dynamic fractional amplitude of low-frequency fluctuations (dfALFF). Then, the occurrence frequency of a dFC or dfALFF state and the co-occurrence frequency of a pair of dFC and dfALFF states among all time points are extracted for each participant to describe their dynamics brain patterns.

**Results:**

We identified a few recurrent states of dfALFF and dFC and further ascertained the co-occurrent patterns of these two types of dynamic brain states (i.e., dfALFF and dFC states). Importantly, the occurrence frequency of a default-mode network (DMN)-dominated dFC state was significantly different between HCs and SCD patients, and the co-occurrence frequencies of a DMN-dominated dFC state and a DMN-dominated dfALFF state were also significantly different between SCD and MCI patients. These two dynamic features were both significantly positively correlated with Mini-Mental State Examination scores.

**Conclusion:**

Our findings revealed novel fMRI-based neural signatures of cognitive decline from recurrent and concurrent patterns of dfALFF and dFC, providing strong evidence supporting SCD as the transition phase between normal aging and MCI. This finding holds potential to differentiate SCD patients from HCs via both dFC and dfALFF as objective neuroimaging biomarkers, which may aid in the early diagnosis and intervention of Alzheimer’s disease.

**Supplementary Information:**

The online version contains supplementary material available at 10.1186/s13195-020-00764-6.

## Background

Recent studies have focused on the early diagnosis of Alzheimer’s disease (AD) due to a lack of effective treatments. Subjective cognitive decline (SCD), which is considered as a risk state for AD [1, 2], has received increased attention. Neuroimaging techniques have been developed for identifying non-invasive biomarkers at early stages of AD. Because disruption of functional connectivity (FC) emerges at the earliest stage of AD, FC has been considered as a potential neural biomarker for early identification of functional alterations related to AD pathophysiology [3]. However, previous studies have mainly focused on static FC (sFC), which is supposed to be stable at rest, despite FC being highly variable during imaging [4–7]. Dynamic FC (dFC) contains information of the brain’s dynamic functional organization and has attracted increased interest over the past several years [8]. Furthermore, dFC correlates closely with cognition and may be a biomarker for dementia. Progressively altered dFC patterns can effectively track cognitive impairment in AD [9], and disruptions in dFC are detected in both mild cognitive impairment (MCI) and AD [10]. Additionally, a previous study has demonstrated that dFC biomarkers may represent useful surrogate outcomes for the development of preclinical targeted therapeutic interventions [11].

Although the important role of dFC in dementia has been gradually recognized, dynamic regional spontaneous activity has not been well explored. Several studies have indicated that low-frequency resting-state functional magnetic resonance imaging (fMRI) activity, as quantified by the amplitude of low-frequency fluctuations (ALFF) or fractional ALFF (fALFF), is well-suited to measure cognitive capabilities [12, 13], but it remains unclear whether the dynamic patterns of ALFF or fALFF are relevant to cognitive decline. Although evidence has shown that regional spontaneous neural activity is closely related to FC [14, 15], little is known in regard to the relationship between dynamic patterns of ALFF/fALFF and FC and whether this relationship is linked to cognitive decline.

In the present study, we investigated recurrent dynamic fALFF (dfALFF) and dFC patterns (i.e., states), as well as the percentage of the time point of each state and the co-occurrence of each pair of these two types of states at all time points from resting-state fMRI recorded in SCD patients, MCI patients, and healthy controls (HCs). We hypothesized that dfALFF and dFC would exhibit a few recurrent and concurrent patterns and that these patterns would be different among HC, SCD, and MCI groups. Thus, these recurrent and concurrent patterns identified from dynamic regional activity and FC may potentially serve as neuroimaging biomarkers for the diagnosis of SCD and the conversion from SCD to MCI.

## Methods

### Subjects

The present sample included 62 SCD patients and 75 MCI patients, as well as 70 HCs matched with SCD and MCI patients by age, gender, and years of education. Table 1 summarizes their demographic data and other relevant characteristics. These individuals were recruited from the First Affiliated Hospital of Guangxi University of Chinese Medicine and from the community and elderly activity centers in Nanning from April 2016 to January 2018. The inclusion criteria for patients were as follows: (1) age between 55 and 75 years, (2) right-handed, and (3) daily-life abilities and social occupations were not affected. The exclusion criteria for patients were as follows: (1) other diseases that were terminal, severe, or unstable; (2) severe hearing or visual impairment; (3) dementia, cerebral infarction, or physical/neurological disorders that could cause brain dysfunction; (4) drugs that may cause cognitive changes or organ failure were administered before inclusion; or (5) fMRI-examination contraindications. To assess the general cognitive and functional status of the included individuals, the following set of screening questionnaires were used: Mini-Mental State Examination (MMSE) [16], Montreal Cognitive Assessment (MoCA) [17], Clinical Dementia Rating (CDR) [18], Geriatric Depression Scale (GDepS) [19], and Global Deterioration Scale (GDS). MCI patients were diagnosed according to the criteria established by a previous study [20] as follows. First, the main complaint was memory impairment and another informed individual confirmed this symptom. Second, other cognitive functions were relatively intact or only slightly impaired. Third, the ability of daily living was not affected. Fourth, the diagnostic criteria of dementia were not met. Fifth, other systemic diseases that could cause a decline in brain function were excluded. Finally, the MMSE score was 24–27, the CDR score was 0.5, and the GDS score was 2–3. SCD and HC groups are determined as follows. First, the MMSE score was > 27, the CDR score was 0, and the GDS score was 1. Second, the following six tests in three cognitive domains (memory, language, and attentive/executive functions): Auditory Verbal Learning Test (AVLT delayed recall and AVLT-recognized) [21], Animal Fluency Test (AFT) [22], 30-item Boston Naming Test (BNT) [23], and Trail Making Test (STT-A and STT-B) [24] were applied. Third, subjects were excluded if any of the following occurred: abnormalities on two measures in the same cognitive domain, defined as > 1 standard deviation (SD); or if each of the three cognitive domains had an impaired score (defined as > 1 SD) [25]. Fourth, individuals who had complained of a declining memory were regarded as the SCD group [26], whereas individuals with no complaints and whose cognitive functions passed neuropsychological tests were included in the HC group. All neuropsychological assessments were completed by two neurologists with more than 5 years of clinical experience. A flowchart of the diagnostic steps in our present study is shown in Fig. S1 of the Supplementary Materials.

**Table 1:** Demographic and neuropsychological data of each group

	HC (***n*** = 66)	SCD (***n*** = 55)	MCI (***n*** = 65)	***p*** value
Age (years)	64.68 ± 5.78	64.47 ± 5.41	64.92 ± 6.68	0.650
Gender (males/females)	66 (24/42)	55 (18/37)	65 (18/47)	0.567
Education (years)	11.76 ± 3.02	12.05 ± 3.08	10.66 ± 2.55	0.242
MMSE	29.11 ± 0.75^c^	28.85 ± 0.85^b^	25.92 ± 1.05^b,c^	10^–33^*
MOCA	26.12 ± 2.06^a c^	24.93 ± 2.26^a b^	21.62 ± 2.73^b,c^	10^–16*^
GDepS	4.17 ± 2.27^c^	4.60 ± 2.61^b^	5.57 ± 2.10^b,c^	0.005*
CDR	0	0	0.5	–

Age, education, MMSE scores, MOCA scores, and GDepS scores were tested via analysis of variance (ANOVA), Kruskal-Wallis tests, two-sample *t* tests, or Mann-Whitney tests. Gender was tested via a chi-squared test

*Significantly different among the three groups (*p* < 0.05, ANOVA)

^a^Significantly different between the HC and SCD groups (*p* < 0.05, two-sample *t* test)

^b^Significantly different between the SCD and MCI groups (*p* < 0.05, two-sample *t* test)

^c^Significantly different between the HC and MCI groups (*p* < 0.05, two-sample *t* test)

### MRI acquisition

The imaging data were scanned using a 3.0-T MRI scanner (Magnetom Verio, Siemens Medical, Erlangen, Germany). The structural MRI data were collected in a sagittal orientation using magnetization-prepared rapid-gradient echo sequences with the following imaging parameters: TR/TE = 1900 ms/2.22 ms, FOV = 250 mm × 250 mm, slice thickness = 1 mm, matrix size = 256 × 256, flip angle = 9°, and number of slices 174 = 176. The resting-state functional MRI data were collected in an axial orientation using multi-slice-gradient echo-planar imaging sequences with the following imaging parameters: TR/TE = 2000 ms/30 ms, FOV = 240 mm × 240 mm, slice thickness = 5 mm, matrix size = 64 × 64, flip angle = 90°, number of slices = 31, and number of volumes = 180. The day before scanning, subjects were asked to ensure sufficient sleep quality and to not drink alcohol or take drugs that might affect the nervous system. During scanning, subjects were instructed to not engage in any particular cognitive or motor activities, keep their eyes closed, relax, and not fall asleep. Foam padding and headphones were used to limit head movement and reduce scanner noise.

### MRI preprocessing

In this study, we used a popularly-used fMRI preprocessing routine, as developed in the Data Processing Assistant for Resting-State fMRI (DPABI, http://rfmri.org/dpabi) [27, 28] and based on some functions in Statistical Parametric Mapping (SPM8, https://www.fil.ion.ucl.ac.uk/spm) [29]. All the preprocessing steps of T1-weighted and resting-state fMRI data were conducted by DPABI. The preprocessing pipeline was as follows. The first five volumes were removed to avoid a T1-equilibration effect, after which 175 volumes remained. The fMRI data consisted of images acquired one slice at a time; thus, each slice was acquired at a slightly different time point. Additionally, motion correction was used to adjust the time series of images so that the brain was in the same position in every image. Hence, we used DPABI to correct for differences in image acquisition time and head position from different slices by calling functions in the SPM. The timings of all slices were matched against the middle slice to ensure timing synchronization. The position of the head in each slice was adjusted to that in the first slice to ensure a fixed position across slices. Additionally, head motion parameters were obtained. The brain size, shape, orientation, and gyral anatomy varied largely across the participants. To enable inter-subject comparisons, MRI slices from each brain were transformed or spatially normalized into a standardized template [30]. The Diffeomorphic Anatomical Registration Through Exponentiated Lie algebra (DARTEL) function [31] in DAPBI was used to transform the functional data from the individual native space to the Montreal Neurological Institute space, and the functional data were resliced (3 × 3 × 3 mm^3^ voxels) and smoothed with a 4-mm FWHM. We further reduced the effects of physiological artifacts of whole-brain signals via a regression analysis in DPABI. In addition to the global mean signal, six motion parameters, cerebrospinal-fluid signals, and white-matter signals were removed as nuisance variables to reduce the effects of head motion and non-neuronal BOLD fluctuations. Before estimating dFC, temporal band-pass filtering (0.01–0.10 Hz) was performed to remove the effects of low-frequency drift and high-frequency noise in DPABI. The choice of ROIs determines the tradeoff between spatial coverage and resolution and should be carefully made. We chose Dosenbach’s ROIs, which are functionally representative to sample the whole brain [32]. Dosenbach’s ROIs have a clear coordinate definition for the location of structural partitions of the whole cerebral cortex and groups the ROIs into six types of networks, namely, the cerebellar, opercular, default, parietal, occipital, and sensorimotor networks. We also added four subcortical ROIs located in the bilateral amygdala and para-hippocampi according to previous studies [33], and these four ROIs were used as additional networks. Hence, we defined a total of 164 ROIs (spheres with a radius of 8 mm each), consisting of seven networks for subsequent whole-brain analysis. Then, we extracted the time series of each ROI by averaging the time courses of all voxels within the ROI. Finally, we divided the whole brain into seven networks: cerebellar, opercular, default, parietal, occipital, sensorimotor, and additional networks.

### Estimation of dynamic fMRI states

Low-frequency (0.01–0.08 Hz) fluctuations (LFFs) of the resting-state fMRI signals have been reported to be of physiological importance [34] and have been suggested to reflect spontaneous neuronal activity [35]. Furthermore, ALFF and its improved version, fALFF [36, 37] are now widely used for characterizing regional patterns of resting-state fMRI. Hence, we calculated the fALFF based on the protocol [38]. More specifically, ALFF was defined as the sum of amplitudes within a specific low-frequency range (0.01–0.10 Hz), while fALFF was defined as the ratio of the ALFF of a given low-frequency band (0.01–0.10 Hz) to the sum of amplitudes across the entire frequency range detectable in a given signal. In the present study, we used the parameter settings (frequency ranges) in the original paper that introduced fALFF [38].

The dynamic patterns in fALFF were characterized by using the sliding-window approach [10], which sliced ROI time courses into several short data segments with a 50-s rectangular window and estimated a dfALFF matrix for each segment. Next, k-means clustering was used to group the dfALFF matrices into a limited number of clusters, which are referred to as “states.” After the dfALFF states were identified, the occurrence frequency of each state for each participant was obtained by calculating the percentage of the corresponding state among all time points. The dynamic patterns in FC were also characterized by using the sliding-window approach with the same parameters as those used for estimating dfALFF. The occurrence frequency of each dFC state for each participant was also obtained by calculating the percentage of the corresponding state among all time points. Since there were 164 ROIs, one dFC matrix at one time point had the dimensionality of 164 × 164 and the number of elements was 26,896. Because of the symmetry of a dFC matrix, we converted the upper triangle of the dFC matrix into a one-dimensional vector with a dimensionality of 13,366 × 1. A total of 151 vectors (i.e., the number of windows or time points was 151) were obtained for each subject and, for all subjects (*N* = 66 + 55 + 65 = 186), there were in total (151 × 186) = 28,086 dFC vectors. The vectors of all subjects were then concatenated, forming a 13,366 × 28,086 dFC matrix for clustering. Similarly, the clustering algorithm was applied to concatenate the dfALFF vectors of all subjects (164 × 28,086).

**Fig. 1:**
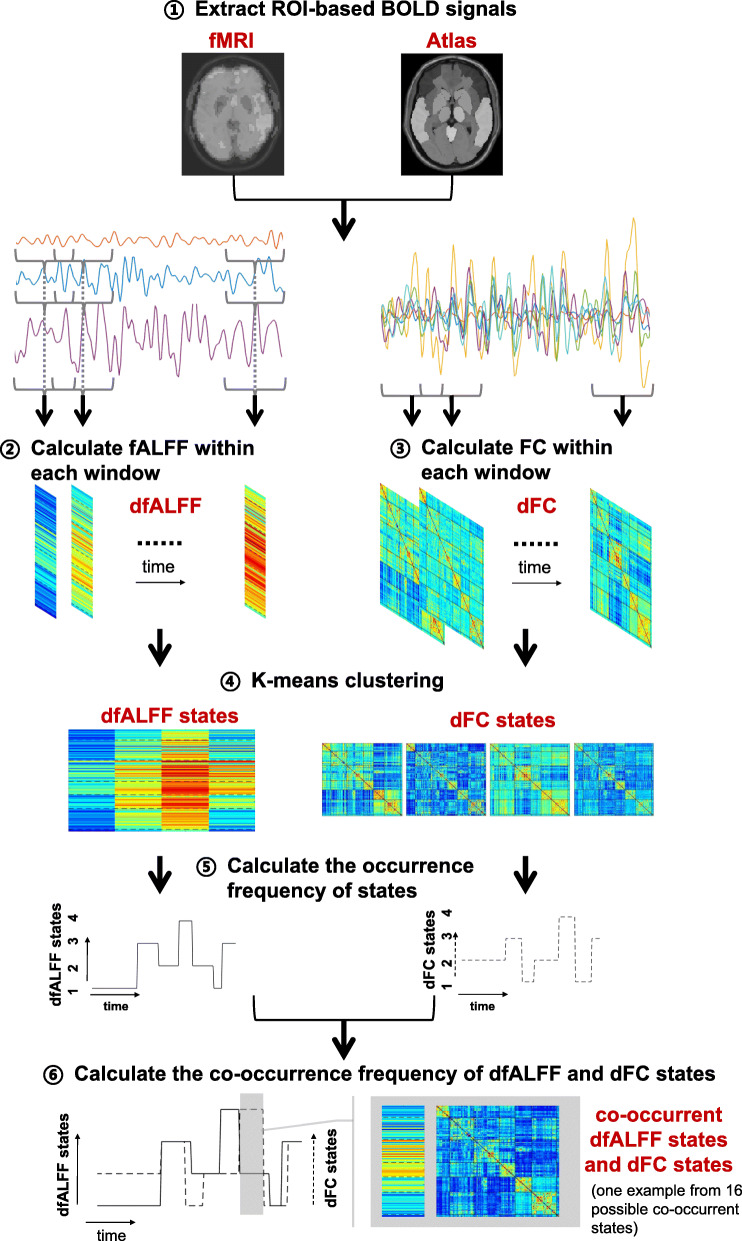
The flowchart for the estimation of dynamic fMRI states. The whole framework for the estimation of dfALFF and dFC states and the calculation of the occurrence/co-occurrence frequency of dfALFF and dFC states

After identifying recurrent states of dALFF and dFC, the co-occurrence frequency between each pair of dfALFF state and dFC state was obtained by calculating the percentage of the co-occurrence of this pair of states among all time points for each participant. The occurrence frequency of a state represents the percentage of a certain dynamic state occurring in the whole timeline, which can be calculated by the ratio of time points with one type of cluster label out of the total time points. The co-occurrence frequency was used to extract regularity of information that occurred simultaneously between two types of dynamic states after identifying their corresponding occurrence frequencies. The co-occurrence frequency of two types of states (e.g., one dFC state and one dfALFF state) represents the percentage of these states occurring simultaneously in the whole timeline, which can be calculated by the ratio of time points with two kinds of cluster labels at the same time point out of the total time points. This entire framework is illustrated in Fig. 1. More details on the estimation of dfALFF and dFC states and their co-occurrence can be found in Appendix A of the Supplementary Materials.

### Statistical analyses

Sociodemographic, clinical, and behavioral variables were tested for normality using the Shapiro-Wilk test. Differences in age, education, MMSE scores, and MOCAscores among the three groups were determined via analysis of variance (ANOVA) or Kruskal-Wallis tests. AVLT, BNT, AFT, and STT differences between the two groups were tested with two-sample *t* tests or Mann-Whitney tests. Gender differences among groups were tested via the chi-squared test. Furthermore, to determine group differences in the functional networks of HCs, SCD patients, and MCI patients, we performed ANOVAs and two-sample *t* tests among the three groups in terms of the occurrence frequencies of dFC states. We used the occurrence frequencies of dFC states 1–4 and dfALFF states 1–4 to perform a one-way ANOVA among the HC, SCD, and MCI groups. The *p* values of eight results (4 dFC states and 4 dALFF states) were corrected for multiple comparisons by using the false discovery rate (FDR) [39]. Based on the significant difference in the occurrence frequency of dFC state 3 among the three groups, we compared the co-occurrence frequency of dFC 3 and dfALFF states 1–4 among HC, SCD, and MCI groups by using one-way ANOVA. The *p* values of four results (4 dfALFF states) were corrected for multiple comparisons by using the FDR. Finally, we conducted Pearson’s correlation analysis to characterize the relationship between dynamic features (the occurrence frequency of dFC states and the co-occurrence frequency between dfALFF states and dFC states) and cognitive scores (MMSE).

## Results

### Sociodemographic and cognitive characteristics

The resting-state fMRI data from 22 participants were excluded due to head motion with more than 2.0-mm maximum displacement in any direction of *x*, *y*, and *z*, or more than 2° of any angular motion throughout the scan. Following these exclusions, data from 55 SCD patients, 65 MCI patients, and 66 HCs remained and were further analyzed. Sociodemographic, clinical, and disease characteristics of the remaining participants are shown in Table 1. Age, education, and the number of participants were not significantly different among the three groups. The MMSE scores were significantly different between SCD and MCI groups, as well as between HC and MCI groups. The MOCA scores were significantly different between any two compared groups. The GDepS scores were significantly different between SCD and MCI groups, as well as between HC and MCI groups.

### Dynamic fMRI states

**Fig. 2:**
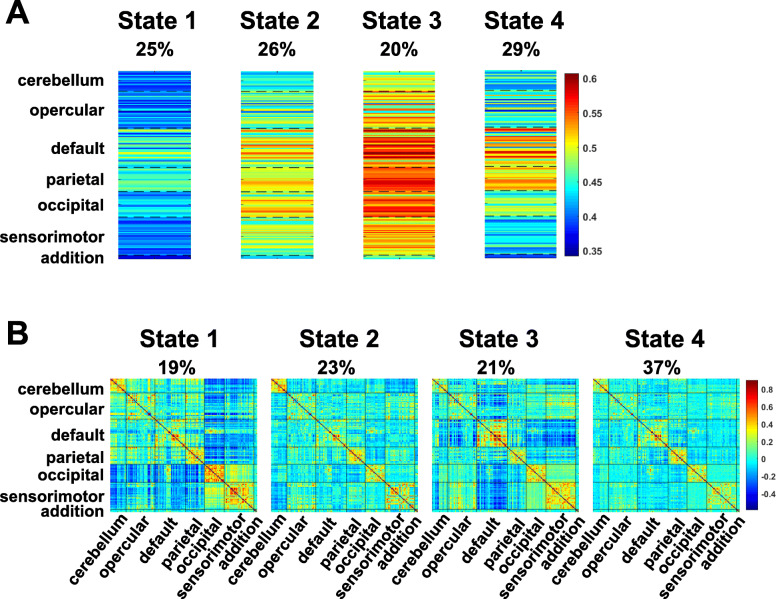
The identified 4 dfALFF states and 4 dFC states. **a** Four dfALFF states were identified and their occurrence frequencies in all time points of all subjects were 25%, 26%, 20%, and 29%, respectively. **b** Four dFC states were identified and their occurrence frequencies in all time points of all subjects were 19%, 23%, 21%, and 37%, respectively

**Fig. 3:**
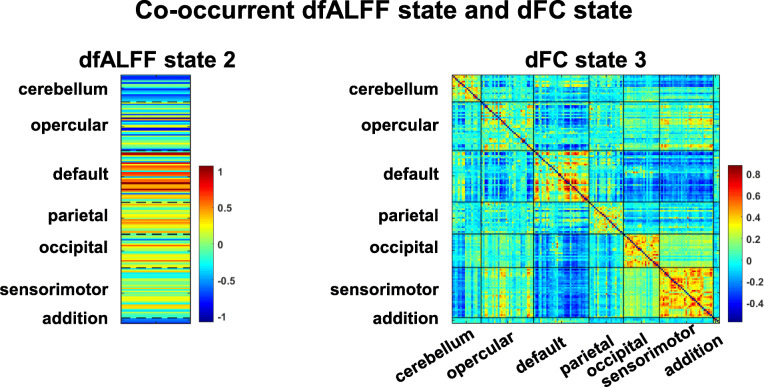
An example of co-occurrent dfALFF state and dFC state. The co-occurrence dfALFF state 2 showed a strongest local activation within DMN, which is consistent with co-occurrence dFC state 3 (a DMN-dominated state)

We identified four dfALFF states and four dFC states (Fig. 2). The results showed that dFC state 3 had the strongest positive within-DMN FC and negative between-DMN FC (Fig. 2b); hence, dFC state 3 was regarded as a DMN-dominated state. One pair of co-occurrence states (dfALFF state 2 and dFC state 3) is shown in Fig. 3. The co-occurrence dfALFF state 2 showed the strongest local activation within the DMN, which is consistent with its co-occurrence with dFC state 3 (a DMN-dominated state). More details on the main characteristics of dfALFF and dFC states can be found in Appendix B of the Supplementary Materials.

### Group differences in dfALFF and dFC states

**Fig. 4:**
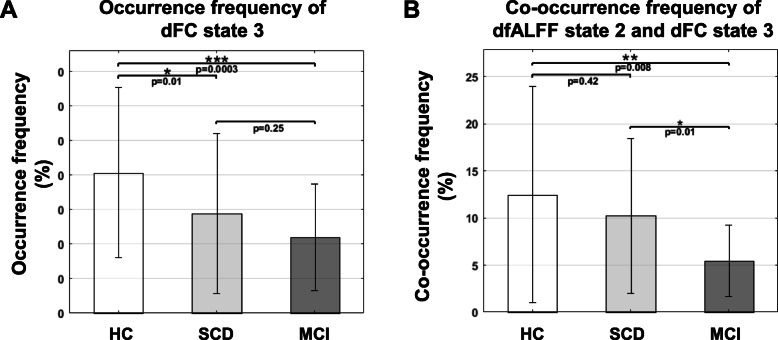
Group difference of dfALFF and dFC states between HC, SCD and MCI. **a** The occurrence frequency of dFC state 3 is significantly different among the three groups. SCD showed significantly reduced occurrence frequency compared with HC (*p* = 0.01), and MCI showed significantly reduced occurrence frequency compared with HC (*p* = 0.0003). **b** The co-occurrence frequency of dfALFF state 2 and dFC state 3 is significantly different among the three groups. MCI showed significantly reduced co-occurrence frequency compared with SCD (*p* = 0.01), and MCI showed significantly reduced co-occurrence frequency compared with HC (*p* = 0.008)

The results of an ANOVA showed that there were significant differences among the three groups in the occurrence frequency of dFC state 3, as well as in the co-occurrence frequencies of dfALFF state 2 and dFC state 3, as shown in Fig. 4 and Table S1 and S2 of the Supplementary Materials. There were no significant differences among the groups in terms of the occurrence frequencies of dfALFF states (Fig. S2 of the Supplementary Materials). Specifically, the SCD and MCI groups showed significantly lower occurrence frequencies of dFC state 3 compared to that of HCs (*p* = 0.01 and *p* 325 = 0.0003, respectively); however, there was no significant difference in the occurrence frequencies of dFC state 3 between the SCD and MCI groups (*p* = 0.25). The MCI group showed significantly reduced co-occurrence frequencies of dfALFF state 2 and dFC state 3 compared to those of the SCD and HC groups (*p* = 0.01 and *p* = 0.008, respectively), whereas there were no significant differences in these co-occurrence frequencies between the SCD and HC groups (*p* = 0.42).

### Correlations between dynamic fMRI states and cognitive scores

**Fig. 5:**
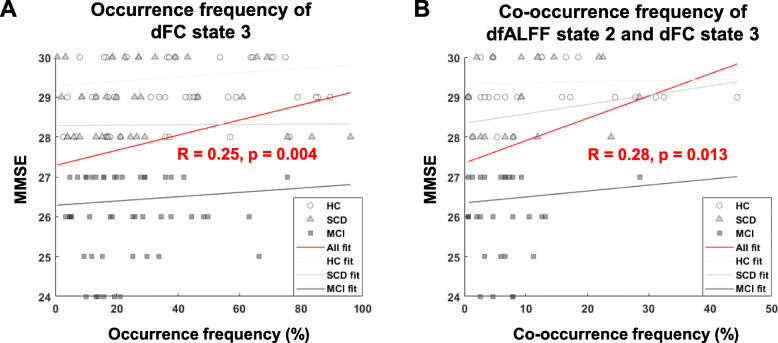
The correlation between dynamic fMRI state features and MMSE scores. **a** The occurrence frequency of dFC state 3 was significantly positively correlated with MMSE (*R* = 0.25, *p* = 0.004). **b** The co-occurrence frequency of dfALFF state 2 and dFC state 3 was significantly positively correlated with MMSE (*R* = 0.28, *p* = 0.013)

The correlations between dynamic fMRI states and cognitive scores are shown in Fig. 5. The occurrence frequency of dFC states 3 was significantly positively correlated with MMSE scores (*R* = 0.25, *p* = 0.004), while the co-occurrence frequencies of dfALFF state 2 and dFC state 3 were significantly positively correlated with MMSE scores (*R* = 0.28, *p* = 0.013).

## Discussion

The present study proposed a novel resting-state fMRI-analysis framework to explore dynamic regional neural activity and FC in SCD and MCI patients. We examined dynamic patterns of FC and fALFF (i.e., dFC and dfALFF) and estimated a few recurring dFC states and dfALFF states. A dFC state is one specific recurring pattern of whole-brain FC, while a dfALFF state is one specific recurring pattern of whole-brain regional spontaneous activities. One dFC/dfALFF state may be related to a specific mental state of subjects at rest. Hence, the occurrence frequency of one dFC or dfALFF state and the co-occurrence frequency of one pair of two types of states are important metrics specific to each subject. We found that dFC state 3 had the strongest positive within-DMN FC and negative between-DMN FC and was consequently regarded as DMN-dominated state. Moreover, the HC, SCD, and MCI groups exhibited different dFC and dfALFF patterns: the occurrence frequencies of a DMN-dominated dFC state were different between the HC and SCD groups, while the co-occurrence frequencies of a DMN-dominated dfALFF state and a DMN-dominated dFC state were different between the SCD and MCI groups.

### Importance of dynamic state analysis

The human brain is connected by overlapping functional networks that present interacting and interdependent relationships with each other to maintain cognitive functions [40, 41]. During resting states, there still exists consistent spontaneous activation and information transmission in the brain [42]. Hence, investigating dynamic brain states can more accurately reflect the resting-state activity and connectivity of the human brain and can provide a more comprehensive understanding of the brain [43]. Dynamic state analysis of the brain has been gradually used to study preclinical stages of AD. It has been suggested that functional dynamic neuroimaging biomarkers are well-suited to detect neural signatures at the earliest preclinical stages of AD, far before measurable changes in neurochemistry, anatomical structure, and/or cognition [44]. A previous study applied eight resting-state measures and found that FC dynamics, as well as ALFF and FC matrices, were most discriminated for AD classification, and that classification accuracy was slightly improved by combining all of these measures [45]. Another study suggested that dFC may represent a more important biomarker of dementia than sFC because its progressively altered patterns can better track cognitive impairment in AD and subcortical ischemic-vascular disease (SIVD) [9]. Furthermore, disruptions in dFC that have been extended to sFC results have been detected in both MCI and AD patients [10]. Homeoplastically, we found a significant decrease in the occurrence frequency of the DMN-dominated dFC state in the SCD and MCI groups compared with that in the HC group. We also found a decrease in the co-occurrence frequency of the DMN-dominated dfALFF state and DMN-dominated dFC state in the MCI group compared with that in the SCD and HC groups. Collectively, these findings may help to further elucidate the pathophysiology of AD and may provide objective neuroimaging biomarkers for the identification of SCD. Particularly, unlike previous related dynamic brain studies only focusing on dFC, this work also investigated the time-varying patterns of regional brain activity (i.e., dfALFF) and proposed a new measure (the co-occurrence frequency of the dfALFF state and dFC state) to characterize the dynamic brain. Because regional brain activity is the source data used to estimate FC, dfALFF and dFC should be related to each other.

However, it still remains unclear how dfALFF states and dFC co-exist and co-evolve and how the co-existence and co-evolutionary patterns are altered in specific cohorts, such as SCD and MCI patients. Because the co-occurrence frequency of the DMN-dominated dfALFF state and DMN-dominated dFC state is correlated with cognitive performance, we speculate that the co-occurrence or co-existence of these two different types of dynamic states (states of regional activities and connectivity) reflects the brain’s capability to maintain strong correlation and synchronization among cognition-related regions and is important to support cognition. Therefore, the aberrant patterns of co-occurring dFC and dfALFF states could be indicative of the decline in cognitive ability and could be a marker of the progression of dementia. The proposed new dynamic brain state analysis method has the capability of revealing the co-existing and co-evolving patterns of two different but correlated dynamic states (dynamic regional activity and dynamic functional connections among local regions), so it is a powerful tool to reveal new and more complete patterns of the dynamic brain. The new analysis method can also be potentially used for the investigation of disrupted and abnormal brain functions, providing new insights into the mechanisms of mental disorders.

### SCD as a transition stage to MCI

Our present results of dynamic-state analyses of fMRI suggest that there is a two-stage progression from normal aging to MCI, in which SCD is a transition stage. In the first stage (from HC to SCD), the brain’s functional abnormality emerged as a decrease in the occurrence of a DMN-dominated dFC state; in the second stage (from SCD to MCI), the brain’s functional abnormality was exhibited as a new pattern, which was represented as a decrease in the co-occurrence of a DMN-dominated dFC state and a DMN-dominated dfALFF state. Therefore, it is possible that the emergence of SCD is related to a change in functional brain networks but may not be related (or is less related) to regional spontaneous activities. Next, regional spontaneous activities may also play an important role in the progression from SCD to MCI. More precisely, the progression to MCI is related to co-occurrent states of regional spontaneous activities and FC. Cognitive decline in the early stage of AD is mainly related to aberrant FC, while cognitive decline in the late stage of AD is related to both aberrant regional activities and FC. Because FC and regional activities play different roles before and after SCD, SCD may represent a transition phase between normal aging and MCI. However, further studies are needed to confirm or refute this hypothesis.

### The role of DMN-dominated states

The significantly altered dynamic states across groups in the present study were dominated by the DMN, both in terms of dFC states and dfALFF states. We found that dFC state 3 was a DMN-dominated state because it had the strongest within-DMN FC. Also, dfALFF state 2, of which the co-occurrence frequency with dFC state 3 was different between MCI and SCD patients, was dominated by the DMN because the DMN had the strongest dfALFF among all networks. The DMN is the core of intrinsic- connectivity networks, of which the corresponding FC is positively correlated with cognitive performance [46] and is also vulnerable to AD [47, 48]. Studies have found variable and complex patterns of altered activity or connectivity of the DMN in MCI [49], and previous studies of DMN hyper-connectivity have suggested functional disconnection and compensation for damage in early AD [47, 50]. As a high-risk state of AD, SCD shares similar patterns of brain abnormalities to those of AD, and the disruption of brain connectivity in SCD is similar to that observed in MCI [51, 52]. Moreover, SCD shows intermediate changes in DMN connectivity between MCI patients and HCs [51, 53]. Analogously, our present study found that SCD showed intermediate changes in DMN-dominated FC/fALFF states. According to the above results, we speculate that enhanced FC of the DMN may lead to a decreased occurrence frequency of the whole-brain DMN-dominated state in order to maintain normal brain function. It is noteworthy that the occurrence frequency of the DMN-dominated dFC state was not significantly different between SCD and MCI groups, implying that disruption of whole-brain network tends to remain relatively stable in the process of conversion from SCD to MCI. Likewise, we observed intermediately decreased co-occurrence frequencies of the DMN-dominated dFC and dfALFF states in the SCD group compared to those in the MCI and HC groups, while there was no significant difference in this co-occurrence frequency between SCD and HC groups. In this regard, we speculate that DMN dysfunction or disconnection occurred in SCD and MCI patients, resulting in whole-brain dynamic network decline despite a predominantly active DMN during the resting state. According to a proposed theoretical framework of cascading network failure of AD in the DMN, high FC may result from high-processing burden, which may be shifted when overloaded and/or during noisy/inefficient synaptic communication. These changes may then spread to downstream regions of highly connected networks as a compensatory strategy and may eventually cause widespread system failure [54]. It has been indicated that dysfunction in one region may result in DMN hyperconnectivity [54], which has been interpreted as a compensatory phenomenon [55]. Similarly, it was found that posterior DMN decline was accompanied by increased connectivity with other brain networks throughout the course of AD [56]. A longitudinal study demonstrated that the connectivity within the anterior and ventral DMN was increased initially but ultimately deteriorated as the disease progressed [57], suggesting that dysfunction of the DMN developed gradually across the AD spectrum and ultimately progressed to become non-functional [58] and/or with gray-matter atrophy [59]. In the present study, we did not observe a significant difference in the co-occurrence frequency between the SCD and HC groups. We speculate that disruption of whole-brain network dynamics revealed by the DMN in SCD was relatively mild and that temporal synchronization of regional neural activity and FC was maintained via compensatory mechanisms. During progression of AD, our data suggest that whole-brain network dynamics became progressively disrupted, as indicated by a decreased co-occurrence frequency of DMN-dominated dfALFF and dFC states in MCI patients.

## Limitations

Our present study had some limitations. Owing to a lack of ad-hoc technology and equipment, we were unable to obtain information regarding amyloidosis, which is an important biomarker of AD. In addition, future longitudinal studies may help to better characterize the progression of AD and provide additional insights into the conclusions of our present study.

## Conclusions

In summary, our present study introduced a novel dynamic-fMRI state-analysis framework for dfALFF and dFC analyses. Our findings provide new insights into the spatiotemporal functional organization of the brain during resting states, as well as a more comprehensive understanding of the roles of regional spontaneous neural activity and FC during cognitive decline. From the evidence of dynamic states of FC and regional activity, SCD may be regarded as a transitional stage between normal aging and MCI, and DMN-dominated states may play an important role in cognitive decline.

## Supplementary Information

**Additional file 1.**

## Data Availability

The datasets used and/or analyzed during the current study are available from the corresponding author on reasonable request.

## Abbreviations

AD: Alzheimer’s disease
AFT: Animal fluency test
ALFF: Amplitude of low-frequency fluctuations
ANOVA: Analysis of variance
AVLT: Auditory Verbal Learning Test
BNT: Boston Naming Test
CDR: Clinical Dementia Rating
dFC: Dynamic functional connectivity
DMN: Default mode network
fALFF: Fractional amplitude of low-frequency fluctuations
FDR: False discovery rate
GDepS: Geriatric Depression Scale
GDS: Global Deterioration Scale
HC: Healthy controls
MCI: Mild cognitive impairment
MMSE: Mini-Mental State Examination
MoCA: Montreal Cognitive Assessment
ROI: Region of interest
SCD: Subjective cognitive decline
SD: Standard deviation
SPM: Statistical Parametric Mapping
STT: Shape Trail Test
TMT: Trail Making Test
