# Supplementary material for: Recurrent and concurrent patterns of regional BOLD dynamics and functional connectivity dynamics in cognitive decline
Source: Alzheimers Res Ther. 2021 Jan 16;13:28. doi: 10.1186/s13195-020-00764-6 (PMC7811744; doi:10.1186/s13195-020-00764-6)
Supplement: Supplementary file 1 — Additional file 1. [file 13195_2020_764_MOESM1_ESM.docx]

Supplementary materials for “**Recurrent and Concurrent Patterns of Regional BOLD Dynamics and Functional Connectivity Dynamics in Cognitive Decline** ”

**Appendix A: dfALFF and dFC States**

1. *Estimation of dfALFF states*

In our present study, the fALFF was used to measure patterns of local neural activity. The dynamic patterns in fALFF were characterized by using the sliding-window approach, which sliced ROI time courses into several short data segments to estimate the dfALFF matrix. A 25-TR (50-s) rectangular window with a 1-TR step of was used, resulting in 151 windows or data segments. The dfALFF matrix had a dimension of 164 × 151, where 164 was the number of ROIs and 151 was the number of windows. Next, k-means clustering was used to group the dfALFF matrices into a limited number of clusters, which were referred to as “states” in dynamic analysis. The centers of states represented recurring patterns, and the occurrence frequency of each state was extracted. More precisely, the dynamic matrices of all participants (combining HC, SCD, and MCI groups) were first concatenated for k-means clustering with the squared Euclidean distance at the group level. Then, k-means clustering was achieved in two steps. In the first step, the initial points of clustering were randomly set, which was then repeated 100 times; the result with the lowest within-cluster sums of point-to-centroid distances was retained. In the second step, the initial points were set as the centroids of the first-step k-means so that the adverse impact of small fluctuations could be avoided. The optimal number of clusters (i.e., the number of dynamic states) was determined by the elbow method, which calculated the ratio between inter-class distance and intra-class distance. In the present study, the number of states was set to four by using the elbow method (see Figure S3). After the dynamic states of dfALFF were identified, the occurrence frequency of each state was obtained by calculating the percentage of the corresponding state among all time points for each participant.

1. *Estimation of dFC states*

FC was used to measure the patterns of coupled neural connectivity. The dynamic patterns in FC were also characterized by using the sliding-window approach with the same parameters as those used for dfALFF estimation. The dFC matrix had a dimension of 164 × 164 × 151, where 164 and 151 were the number of ROIs and the numbers of windows. Owing to the symmetry of the dFC matrix, the subsequent analysis only used the upper triangle of the matrices at each time point. Then, k-means clustering with the same parameters as those used in the dfALFF estimation was used to obtain the dynamic states for FC. The occurrence frequency of each dFC state was also obtained by calculating the percentage of the corresponding state among all time points for each participant.

1. *Co-occurrence of dfALFF and dFC states*

After identifying reoccurring patterns of dALFF and dFC, the co-occurrence frequency between any two types of states was obtained by calculating the percentage of the two types of states occurring at the same time among all time points for each participant. In the time point where dfALFF states and dFC states coexisted, the co-occurrence dfALFF states and co-occurrence dFC states were represented by the average. Notably, in the process of calculating the occurrence frequency and the co-occurrence frequency, only subjects with at least one window belonging to the state were used.

**Appendix B: Primary characteristics of dfALFF and dFC States**

1. *The main characteristics of the four dFC states were as follows:*

• dFC state 1: strong positive FC within occipital and sensorimotor networks, and strong negative FC between occipital and sensorimotor networks;

• dFC state 2: stronger positive FC within additional networks;

• dFC state 3: stronger positive FC within the DMN, and negative FC between DMN and other networks;

• dFC state 4: weaker whole-brain FC.

1. *The main characteristics of the four dfALFF states were as follows:*

• dfALFF state 1: relatively stronger whole-brain activity, mainly within the DMN, parietal, and occipital networks;

• dfALFF state 2: relatively modest whole-brain activity;

• dfALFF state 3: stronger activity in the DMN, parietal, and occipital networks, and weaker activity in the cerebellar, opercular, sensorimotor, and additional networks;

• dfALFF state 4: the weakest whole-brain activity among all states, mainly within the DMN, parietal, and occipital networks.

# Supplementary Tables

Table S1. Comparisons of occurrence frequencies of dFC and dfALFF states among the HC, SCD, and MCI groups.


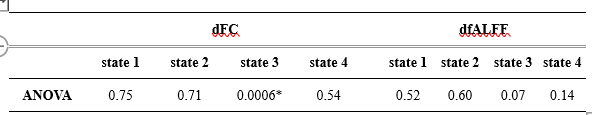


*p-value < 0.05 (FDR corrected)

Table S2. Comparisons of co-occurrence frequencies of dFC state 3 and dfALFF states among the HC, SCD, and MCI groups.


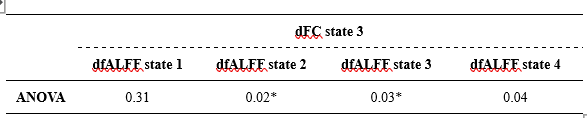


*p-value < 0.05 (FDR corrected)

# Supplementary Figures


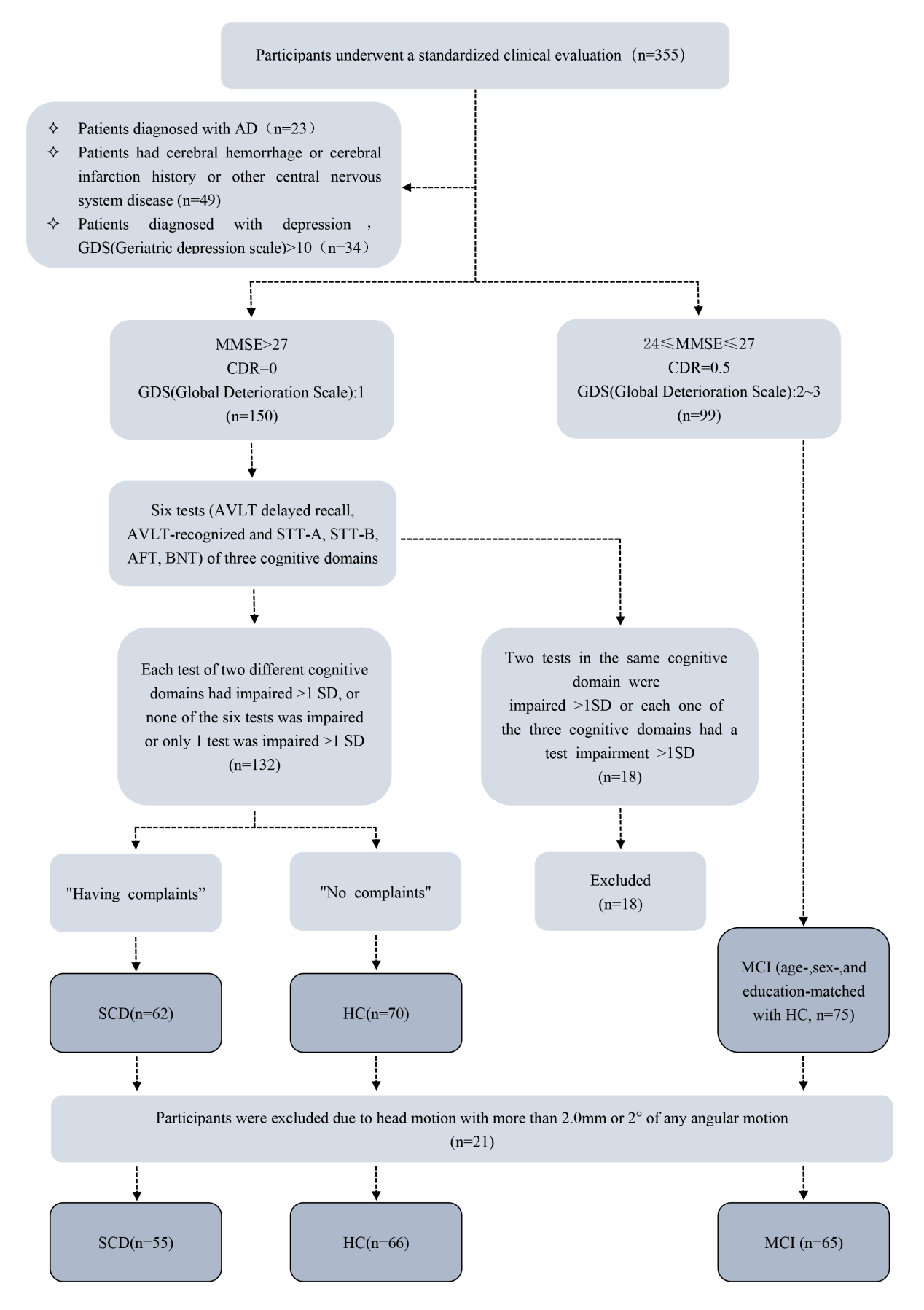


Figure S1. Flowchart of subject diagnostic criteria.


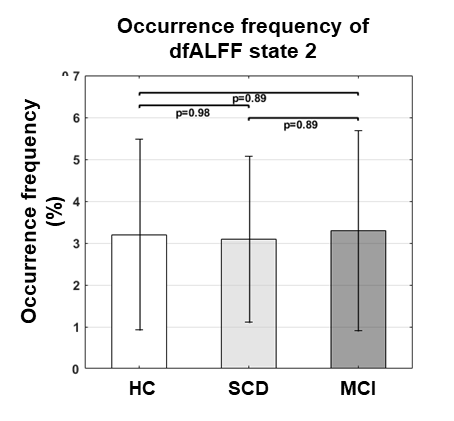


Figure S2. Group differences in dfALFF states among the HC, SCD and MCI groups.


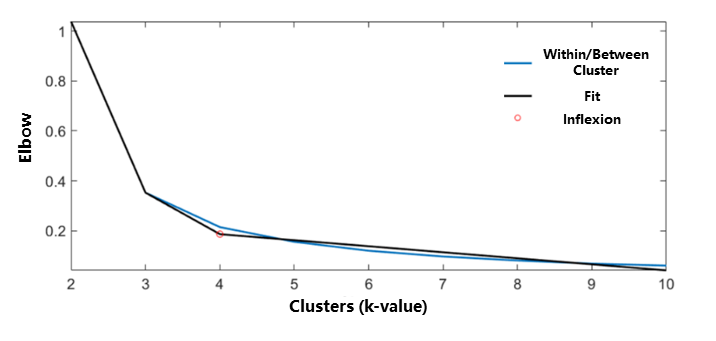


Figure S3. The dFC clustering results using different numbers of clusters, k-value, in k-means. The optimal k=4 (indicated by the red circle) was selected by the elbow method.
